# Immune responses in patients with HIV infection after vaccination with recombinant Hepatitis B virus vaccine

**DOI:** 10.1186/1471-2334-6-65

**Published:** 2006-03-30

**Authors:** Neelam Pasricha, Usha Datta, Yogesh Chawla, Surjit Singh, Sunil K Arora, Archana Sud, Ranjana W Minz, Biman Saikia, Haqeeqat Singh, Isaac James, Shobha Sehgal

**Affiliations:** 1 Department of Immunopathology, Post Graduate Institute of Medical Education and Research, Chandigarh, India; 2 Department of Hepatology, Post Graduate Institute of Medical Education and Research, Chandigarh, India; 3 Department of Internal Medicine, Post Graduate Institute of Medical Education and Research, Chandigarh, India

## Abstract

**Background:**

Patients with HIV infection are at risk of co-infection with HBV, as the routes of transmission are shared and thus immunization with HBV vaccine could be protective in them. The aim of the present study was to assess the efficacy of recombinant vaccine in treatment-naive HIV positive patients and healthy controls, and to dissect out differences if any, in different limbs of immune response.

**Methods:**

Forty HIV positive patients and 20 HIV negative controls, negative for HBsAg, HBsAbs and HBcAbs were vaccinated with three doses of 40μg and 20μg of vaccine respectively. Patients were divided into high CD4 and low CD4 group based on CD4+ lymphocytes of 200 and < 200/mm3 respectively. Group II consisted of healthy controls. Detection of phenotypic markers was done by flowcytometry. Cytokine estimation was done by sandwich ELISA. HBsAbs were estimated in serum by ELISA.

**Results:**

After vaccination, CD_4_+, CD_8_+ and CD_3_+ cells increased significantly in all the groups. There was no increase in NK cell activity in patients with high CD_4_+ lymphocytes and only a marginal increase in patients with low CD_4_+ lymphocytes (170 to 293/mm3) whereas a marked increase was observed in controls (252 to 490/mm3). After vaccination, although an increase in memory cells was observed in HIV positive patients, yet HBsAb levels were significantly lower than controls (P < 0.05) indicating a functional defect of memory cells in HIV/AIDS patients. Basal IFN-γ levels were also significantly lower in HIV/AIDS patients (P < 0.01). Although the levels increased after vaccination, the peak level remained lower than in controls. HBsAb titers were much lower in HIV positive patients compared to controls. (High CD_4_+ group: 8834 mIU/ml, low CD_4_+ group: 462 mIU/ml Vs. Controls: 16,906 mIU/ml). IL-4 and IL-10 were low in patients.

**Conclusion:**

Despite a double dose in patients, IL-4 and IL-10, which regulate antibody response, were also lower in patients, and this together with low CD_4_+ counts and lack of T help, accounted for low HBsAb levels. Vaccination in patients with CD_4_+ lymphocytes < 50/mm^3^ was ineffective. Thus early immunization is advocated in all HIV positive patients at a stage when they are still capable of mounting an adequate immune response

## Background

By the end of year 2004 nearly 5.1 million people were living with HIV/AIDS in India [1]. Patients with HIV infection are frequently co-infected with hepatitis B virus (HBV) as routes of transmission are shared. The prevalence of HBV co-infection depends on the prevailing risk factor for acquiring infection in a given population e.g., Out of 30 HIV positive homosexuals from Maryland USA, 23 were positive for HBsAbs, 24 for antiHBc and only three patients were negative for all the hepatitis B markers [2]. A preliminary study at our institute documented HBcAbs in 22 out of 80 patients, 6 were HBsAg positive and four patients had evidence of replicating virus [3].

Acute hepatitis caused by HBV is milder in HIV infected patients but chronic disease is more frequent with a poorer prognosis and increased infectivity [4]. Patients who have HIV before their HBV infections are more likely to become HBsAg carriers. On the other hand, prior HBV infection is unlikely to be specifically associated either with acquisition of HIV infection or increased rate of progression [5]. The development of HBV vaccines has offered new strategies for protecting these high-risk groups. There are conflicting reports of antibody response against HBV vaccine in HIV positive patients depending upon the stage of the disease.

Although substantial literature is available regarding antibody response to different HBV vaccines in Caucasians, yet precise events pertaining to cellular immune responses, which are crucial for an optimal immune response are either sparse or their correlation with antibody response is imperfect. Further, since immune responses are linked to environmental, genetic and nutritional factors, these are likely to vary in different geographical areas in different clades and different population groups. Thus, data obtained from the western countries cannot be reliably extrapolated to Indian population. The present study was aimed to assess the efficacy of an indigenous HBV vaccine in HIV positive patients harboring mainly clade C [6] and to study the different cell populations with their functional attributes especially in relation to HBsAb formation. An insight into various cellular subsets and immune aberrations in HIV positive patients in response to HBV vaccination are expected to give valuable leads for better planning and evaluation of other vaccines which may be required in a rapidly expanding HIV infected population.

## Methods

Diagnosis of HIV was established as per National AIDS Control Organization (NACO) guidelines (WHO criteria adopted by NACO) [7]. HIV infected, asymptomatic patients attending special medical out patient department of PGIMER, Chandigarh (India) were enrolled in the study. These patients could not afford Highly Active Anti-Retroviral Treatment (HAART). Control subjects were recruited from the healthy HIV negative hospital staff. Written consent was obtained and trained counselors performed pre-test counseling.

HIV infected patients and controls were screened for HBsAg and HBsAb by ELISA using Monalisa HBsAg and Monalisa antiHBs 3.0 kits (Sanofi Diagnostics, Pasture). Subjects positive for HBsAg, HBsAb and HBcAb were excluded from the study. A total of 40 asymptomatic HIV positive patients and 20 control subjects were included in the vaccination and immune assessment study under 2 groups. HIV positive patients were subdivided into two groups on the basis of their CD_4_^+ ^lymphocytes, group IA, high CD_4 _group with an absolute CD_4_^+ ^lymphocyte count of ≥ 200/mm^3 ^and group 1B, low CD_4 _group with an absolute CD_4_^+ ^lymphocyte count of < 200/mm^3^. Group II consisted of 20 normal healthy control subjects.

### Vaccine dosage and administration

All the patients and controls received three doses of recombinant Senvac™ HBV vaccine (Shanta Biotechnics, Hyderabad) in the deltoid region at 0, 1 and 6 months. All HIV positive patients were given 40 μg (double dose) of vaccine in each dose, while controls subjects received 20 μg (usual dose) of vaccine.

### Immune assessment

*Phenotypic characterization *of immune cells was done by flowcytometry on EDTA blood using FACSCAN flowcytometer with cell quest software (Becton Dickinson, Immunoflowcytometry System. San Jose, CA, USA). CD_4_^+ ^and CD_8_^+ ^lymphocytes (helper/cytotoxic) were studied using Simultest CD_4_/CD_8 _monoclonal antibodies conjugated with fluorescein isothiocyanate (FITC)/phycoerythrin (PE) respectively. CD_3_^+ ^T cells (pan-T cell) and CD_16+56 _NK (natural killer) cells were studied using Simultest CD_3 _-FITC, CD_16+56 _-PE monoclonal antibodies. Naïve T lymphocytes were studied using CD_45RA_-FITC monoclonal antibodies and memory cells were enumerated using CD_45RO _-PE monoclonal antibodies. The samples for flowcytometry were processed according to the method of Jackson and Warner [8].

*Functional characterization *of TH_1 _and TH_2 _type of T cells was performed on heparinised venous blood according to the method of Luty *et al *[9] with minor modifications. Briefly, leukocyte rich plasma was prepared from 4 ml of heparinised blood and lymphocyte count was adjusted to 3.6 × 10^5^/200 μl. Cultures were stimulated with 10% phytohemagglutinin [PHA-P (DIFCO)] in RPMI-1640 medium (Sigma) and without PHA-P (unstimulated). Cultures were incubated for 20 hrs at 37°C in an atmosphere of 5% CO_2_. The samples were centrifuged at 2000 rpm for 5 minutes and the supernatant stored at -70°C until required for cytokine assay. Cytokines (IL-4, IL-10, IL-12, IFN-γ) were estimated in the culture supernatants by sandwich ELISA using reagents from Pharmingen, (USA) as per manufacturer's instructions. Briefly, culture supernatants were distributed in ELISA plates coated with corresponding anti-cytokine antibodies. Corresponding detector antibodies were used to detect cytokine anti-cytokine complexes. The reaction was developed with TMB in 0.1 M sodium acetate solutions and H_2_O_2_, OD was recorded at 450 nm. The concentration of the cytokines in culture supernatants were calculated from the standard curve for each cytokine plotted on a log-log paper. Functional characterization of B cells was done by estimation of sequential levels of HBsAb by ELISA.

**Figure 1:**
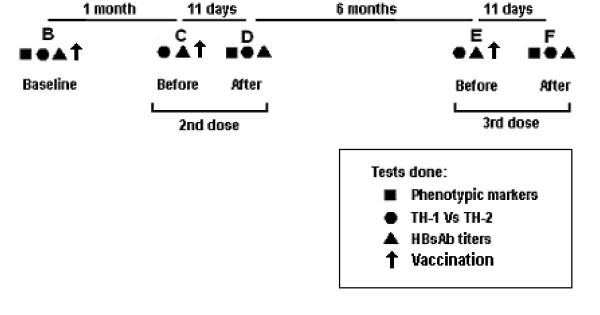
Schematic representation of tests conducted with time schedule.

Phenotypic markers were studied at baseline (B) and 11 days after second (D) and the third (F) dose of vaccine. Functional characterization of TH_1 _and TH_2 _subsets of T helper cells and levels of HBsAbs were estimated at baseline (B), before and 11 days after second (C & D) and before and 11 days after the third (E & F) dose of vaccine. The timing was selected to pick up the peak titers (Figure 1)

## Results

Out of 27 patients with high CD_4 _count, 17 were males and 10 were females and the age ranged between 22 to 45 years with mean age of 30 ± 6 years. Out of 13 patients with low CD_4 _counts, 6 were males and 7 were females and the age ranged between 18 – 50 years with mean age of 32 ± 8 years. Of the 20 normal controls, 12 were males and 8 were females and the age ranged between 18 – 45 years with mean age of 29 ± 7 years.

### T helper (CD_4_^+^) Lymphocytes

**Figure 2:**
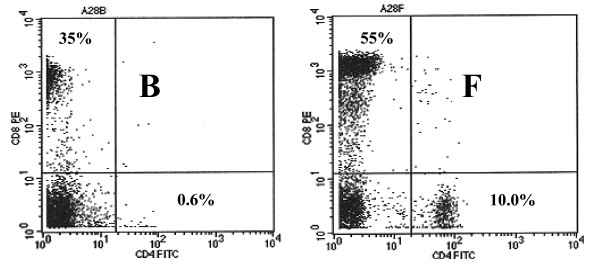
Flowcytometric analysis of CD_4_/CD_8 _lymphocytes in patient with low CD_4 _counts. FL 1 shows FITC labeled CD_4_^+ ^T lymphocytes. FL 2 shows PE labeled CD_8_^+ ^T lymphocytes. Note an increase of CD4 ^+ ^lymphocytes from 0.06% at stage B to 10% at stage F.

**Figure 3:**
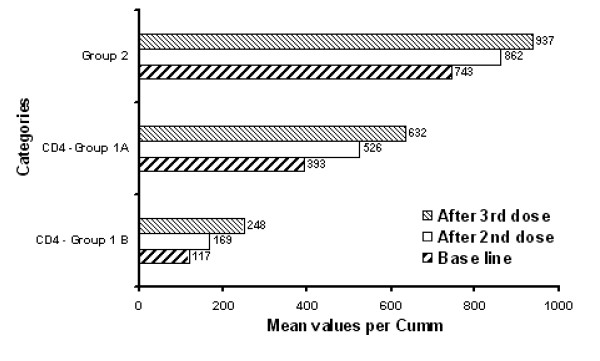
Comparative mean CD_4 _lymphocyte counts between the various groups.

At base line, mean CD_4_^+^lymphocyte counts were highest in controls followed by patients with high CD_4 _counts and those with low CD_4 _counts in that order (p < 0.01; Fig 2B; Table 1). After the second and the third dose of vaccination, 92% and 81% of high CD_4 _group and 61% and 76% of low CD_4 _group patients respectively, showed a statistically significant increase in the number of CD_4_^+ ^lymphocytes (Fig 2 and 3; Table 1).

**Table 1:** Pre and Post vaccination mean of phenotypic markers at various stages in different groups:

	**CD4**	**CD8**	**CD^16+56^**	**CD45^RA^**	**CD 45^RO^**
**Group IA**					
Stage B	393 ± 137/mm^3^	1374 ± 477/mm^3^	292 ± 194/mm^3^	1237 ± 480/mm^3^	1320 ± 490 mm^3^
Stage D	526 ± 178/mm^3^	1580 ± 478/mm^3^	269 ± 139/mm^3^	1471 ± 435/mm^3^	1548 ± 502 mm^3^
Stage F	632 ± 235/mm^3^	1645 ± 408/mm^3^	302 ± 188/mm^3^	1719 ± 590/mm^3^	1727 ± 538 mm^3^
*Statistical analysis*	*B/D p < 0.01*	*B/D p < 0.05*	*B/D p > 0.05*	*B/D p < 0.01*	*B/D p < 0.01*
	*B/Fp < 0.01*	*B/F p < 0.01*	*B/F p > 0.05*	*B/F p < 0.01*	*B/F p < 0.01*
	*D/Fp < 0.01*	*D/F p > 0.05*	*D/F p > 0.05*	*D/Fp < 0.01*	*D/F p < 0.05*
**Group I B**					
Stage B	117 ± 47/mm^3^	1082 ± 828/mm^3^	170 ± 119/mm^3^	760 ± 299/mm^3^	1015 ± 660 mm^3^
Stage D	169 ± 111/mm^3^	1354 ± 1003/mm^3^	193 ± 124/mm^3^	908 ± 341/mm^3^	1125 ± 780 mm^3^
Stage F	248 ± 146/mm^3^	1609 ± 949/mm^3^	293 ± 239/mm^3^	1328 ± 662/mm^3^	1461 ± 799 mm^3^
*Statistical analysis*	*B/D p < 0.05*	*B/D p > 0.05*	*B/D p > 0.05*	*B/D p > 0.05*	*B/D p > 0.05*
	*B/F p < 0.01*	*B/F p < 0.05*	*B/F p < 0.05*	*B/F p < 0.01*	*B/F p < 0.05*
	*D/Fp < 0.01*	*D/Fp > 0.05*	*D/Fp > 0.05*	*D/Fp < 0.05*	*D/Fp < 0.01*
**Group II**					
Stage B	743 ± 208/mm^3^	756 ± 325/mm^3^	252 ± 121/mm^3^	1213 ± 467/mm^3^	1041 ± 398 mm^3^
Stage D	862 ± 263/mm^3^	865 ± 390/mm^3^	364 ± 190/mm^3^	1338 ± 624/mm^3^	1235 ± 303 mm^3^
Stage F	937 ± 183/mm^3^	954 ± 335/mm^3^	490 ± 304/mm^3^	1592 ± 418/mm^3^	1209 ± 256 mm^3^
*Statistical analysis*	*B/D p > 0.05*	*B/D p > 0.05*	*B/D p < 0.05*	*B/D p > 0.05*	*B/D p < 0.01*
	*B/F p < 0.01*	*B/F p < 0.01*	*B/F p < 0.01*	*B/F p < 0.01*	*B/F p < 0.05*
	*D/Fp > 0.01*	*D/Fp > 0.05*	*D/F p < 0.05*	*D/Fp < 0.05*	*D/Fp > 0.05*

Similarly, 65% and 85% of normal control subjects showed an increase in the number of CD_4_^+ ^lymphocytes after the second and the third dose of vaccination. The increase was however more impressive after the third dose of vaccination (Fig 3, Table 1).

### T Cytotoxic CD_8_^+ ^Lymphocytes

At baseline, patients showed a higher number of CD_8_^+ ^lymphocytes as compared to normal subjects (p < 0.01) (Table 1). After HBV vaccination, those with high CD_4 _count showed a statistically significant increase in mean CD_8_^+ ^lymphocytes after each dose of vaccination. While patients with low CD_4 _count showed a statistically significant increase only after the third dose. Normal controls also showed increased mean of CD8^+ ^lymphocytes after third dose of vaccination. There was a gross inversion of the CD_4_/CD_8 _ratio in patients before and after immunization while in controls, the ratio was one. As CD_4_^+ ^and CD_8_^+ ^lymphocytes increased after vaccination, as expected, CD_3_^+ ^lymphocytes also increased after HBV vaccination.

### Natural Killer cells (NK cells)

Basal CD16^+^56^+ ^NK cell number was significantly lower in patients with low CD_4_^+ ^lymphocytes than in controls. After vaccination, patients with high CD_4_^+ ^lymphocytes showed no significant increase in number of NK cells whereas patients with low CD_4_counts responded after the last dose, (p < 0.05). In controls, the NK cells increased significantly after each dose of vaccination (Table 1).

### CD_45RA_^+ ^Naïve cells

**Figure 4:**
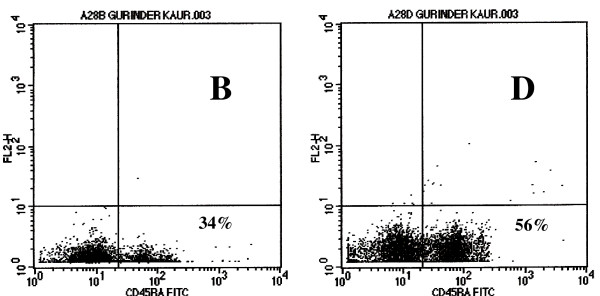
Flowcytometric analysis of CD_45RA_^+ ^Naïve T cells in patient with low CD_4 _counts. FL 1 shows FITC labeled CD_45RA_^+ ^naïve T lymphocytes. Note an increase of CD45RA ^+ ^naïve T cells from 34% at stage B to 56% at stage D.

At baseline, there was no statistically significant difference in CD_45RA_^+ ^cells between controls and patients with high CD_4_^+ ^lymphocytes. Patients with low CD_4 _counts showed a lower number of CD_45RA_^+ ^naïve cells as compared to controls and patients with high CD_4 _counts (p < 0.01). After vaccination, patients with high CD_4 _counts showed a significant increase in CD_45RA_^+ ^naïve cells after each dose of vaccination whereas patients with low CD_4 _counts and normal control subjects showed increase in CD_45RA_^+ ^naïve cells after the third dose of vaccination (Fig. 4, Table 1).

### CD_45RO_^+ ^Memory cells

Memory cells are crucial in response to any immunization. In this study, patients with high CD_4 _counts showed a significantly higher number of CD_45RO_^+ ^memory cells at baseline as compared to controls while patients with low CD_4 _counts showed a lower number of memory cells but the difference between both the patient groups was statistically insignificant. Memory cells also increased after immunization and were higher in HIV positive patients although control subjects showed an early rise of memory cells after vaccination (Table 1).

### HBsAb titers

After vaccination, 40% of patients with high CD_4 _counts formed antibodies at stage C (205 ± 613 mIU/ml; p > 0.05) but 77% of patients had statistically significant levels of HBsAb at stage D i.e. 11 days after second dose (1181 ± 2912 mIU/ml, p < 0.05). Subsequently 46% of patients had transient lowering of HBsAb levels at stage E (524 ± 1478 mIU/ml p > 0.05). After third dose of vaccination at stage F, 100% of patients with high CD_4 _counts showed statistically significant levels of HBsAb [8034 ± 14136 mIU/ml, p < 0.01, (Table 2)]. HBsAb, titers above 100 mIU/ml were considered significant.

**Table 2:** Post vaccination sequential anti HBsAb titers in different groups

**Stages**	**Mean ± SD in group 1A patient**	**Mean ± SD in group 1B patients**	**Mean ± SD in group II normal controls**
Baseline B	0.00 ± 0.00	0.00 ± 0.00	0.00 ± 0.00
Before 2^nd ^dose (C)	205 ± 613	1 ± 4	0.00 ± 0.00
After 2^nd ^dose (D)	1181 ± 2912	26 ± 49	118 ± 397
Before 3^rd ^dose (E)	524 ± 1478	40 ± 90	225 ± 317
After 3^rd ^dose (F)	8834 ± 14136	462 ± 814	16906 ± 21301
**Statistical Significance**	**p**	**p**	**p**
	B/C > 0.05, BD < 0.05	B/C > 0.05 B/D > 0.05	B/C > 0.05 B/D > 0.05
	B/E > 0.05 B/F < 0.01*	B/E > 0.05 B/F < 0.05	B/E < 0.01* B/F < 0.01*

In comparison, only 47% of patients who had a CD_4 _count of < 200/mm^3 ^could form HBsAb titers above 100 mlU/ml. Thirty eight percent of patients could not form antibodies at all, and 15% of patients showed HBsAb titers of < 20 mIU/ml. Thus only 47% of these patients were responders, but with significantly lower HBsAb titers (462 ± 814 mIU/ml; p < 0.5).

The response to vaccination was delayed in normal control subjects. This could be due to the different dosage schedule in patients vs. controls, the former being vaccinated with a double dose. At stage E, 85% of controls formed statistically significant levels of HBsAbs. At final stage, 90% of normal control subjects were responders and there was a striking rise of HBsAb after the last dose. Although in controls 10% were non-responders compared to patients with high CD_4 _counts where 100% of patients responded, yet the mean peak titers (60000 mIU/ml) were much higher in controls than in patients with high CD_4 _counts (Table 2).

### TH 2- Cytokine changes

Basal IL-4 levels were higher in HIV positive patients as compared to IL-4 levels in controls. Although IL-4 levels were higher in patients with high CD_4 _counts than patients with low CD_4 _counts yet the difference was statistically insignificant (p > 0.05) (Table 3). HBV vaccination however failed to elicit adequate IL-4 in HIV positive patients. In controls there was massive increase in IL-4 levels after the first dose of vaccination and levels were much higher at all four stages (C to F) compared to HIV positive patients (Table 3).

**Table 3:** Pre & Post-vaccination mean of IL-4, IL-10 and IFN-γ levels with PHA stimulation at various stages in different groups.

	**Stimulated mean ± SD pg/ml**					**Statistical analysis**
**IL-4**	Baseline (B)	Before 2^nd ^dose (C)	After 2^nd ^dose (D)	Before 3^rd ^dose (E)	After 3^rd ^dose (F)	
Group IA	81 ± 82	95 ± 130	82 ± 55	70 ± 51	81 ± 92	B/C p > 0.05 B/D p > 0.05 B/E p > 0.05 B/F p > 0.05
Group IB	63 ± 69	38 ± 29	48 ± 28	58 ± 62	87 ± 146	B/C p > 0.05 B/D p > 0.05 B/E p > 0.05 B/F p > 0.05
Group II	41 ± 72	284 ± 232*	185 ± 159*	172 ± 151*	810 ± 203*	B/C p < 0.01* B/D p < 0.01* B/E p < 0.01 * B/F p < 0.01*
**IL-10**						
Group IA	162 ± 152	118 ± 94	155 ± 148	160 ± 184	188 ± 194	B/C p > 0.05 B/D p > 0.05 B/E p > 0.05 B/F p > 0.05
Group IB	117 ± 141	70 ± 66	92 ± 75	138 ± 112	139 ± 99	B/C p > 0.05 B/D p > 0.05 B/E p > 0.05 B/F p > 0.05
Group II	295 ± 151	365 ± 213	231 ± 223	229 ± 226*	302 ± 232*	B/C p < 0.05* B/D p < 0.05* B/E p < 0.05 * B/F p < 0.05*
**IFN-γ**						
Group IA	107 ± 106	125 ± 208	133 ± 173	246 ± 255*	274 ± 268*	B/C p > 0.05 B/D p > 0.05 B/E p < 0.01* B/F p < 0.01*
Group IB	72 ± 86	62 ± 131	39 ± 41	154 ± 221*	197 ± 285*	B/C p > 0.05 B/D p > 0.05 D/E p < 0.05* D/F p < 0.05*
Group II	348 ± 216	408 ± 247	292 ± 236	409 ± 304	273 ± 213	B/C p > 0.05 B/D p > 0.05 B/E p > 0.05 B/F p > 0.05

Basal IL-10 levels were also significantly higher in normal control subjects as compared to HIV positive patients and even after vaccination, IL-10 levels remained significantly higher in control subjects as compared to HIV positive patients (Table 3). HBV vaccination thus failed to elicit adequate IL-4 or IL-10 stimulation in HIV infected patients.

### TH 1- Cytokine changes

Basal IL-12 levels were almost similar in control subjects and HIV positive patients. After vaccination, all patients showed delayed but significant increase in IL-12 levels whereas in controls there was massive though transient increase in IL-12 levels (Table 3).

Baseline IFN-γ levels were much higher in control subjects as compared to HIV positive patients (p < 0.01) but the difference in basal IFN-γ levels in patients with high and low CD_4 _counts was insignificant. However after vaccination, levels increased significantly in patients after 6 months. In controls, no further increase of IFN-γ levels could be registered. Yet levels were significantly higher than in patients (Table 3).

## Discussion

It is known that HIV infection may modify the natural history of HBV infection and HIV positive patients have higher rate of HBV chronicity, higher HBV replication and low rates of sero-conversion to anti-HBe and anti-HBs [10,11]. Immunization of HIV positive asymptomatic patients with HBV vaccine could thus protect them from HBV infection and reduce morbidity significantly.

### T cell changes after HBV vaccination

Decreased absolute CD_4_^+ ^lymphocyte number, percentage and decreased CD_4_: CD_8 _ratio occurs early during the course of HIV infection and are predictors of disease progression [12]. We analyzed basal CD_4_^+ ^lymphocytes and changes if any, in the number of CD_4_^+ ^lymphocytes after HBV vaccination. The basal CD_4_^+ ^cell count in Indians were found to be lower than Caucasians (743± 214). Response to any vaccine is dependant on adequate function of CD_4_^+ ^helper cells. In the present study also, response to HBV vaccination was directly correlated with CD_4_^+ ^lymphocyte number. After the second dose of HBV vaccination, 92% of patients with high CD_4 _counts, showed a significant increase in the number of CD_4_^+ ^lymphocytes (peak of 1135/mm^3^) compared to only 61% of patients with low CD_4 _counts (peak of 472/mm^3^). In control subjects, the increase was more impressive after the third dose and peak levels were much higher (1531/mm^3^). It is important to note that mean CD_4_^+ ^lymphocytes increased significantly after vaccination at each stage irrespective of the disease status.

CD_8_^+ ^lymphocytes are responsible for eliminating virus infected cells and act as a second line of defense by becoming activated and eliminating any infected cell, despite the presence of neutralizing antibodies [13]. They can inhibit viral replication by secreting T lymphocyte antiviral factor (CAF), which inhibits RNA transcription [14]. However, there is a paucity of data regarding qualitative and quantitative changes in CD_8_^+ ^lymphocytes after HBV immunization. In the present study, average number of total circulating CD_8_^+ ^lymphocytes in control subjects was 756/mm^3^, which is higher than the figures (500/mm^3^) reported in Caucasians [12]. Mean value of CD_8_^+ ^cells was much higher in HIV positive patients. After immunization CD_8_^+ ^T lymphocytes increased further in all the groups. It has earlier been documented that HIV is associated with a functional defect of CD_8_^+ ^cells. Wilson *et al *[15] had reported that decreased responsiveness to HBV vaccine in HIV infected subjects is associated with elevated CD_8_^+ ^CD_38_^+ ^HLA DR^+ ^T cells. In murine studies it has been observed that repeated exposure to unrelated pathogens, as happens in HIV infected patients, may adversely affect viral specific CD_8_^+ ^lymphocytes [16].

At baseline, CD_4_:CD_8 _ratio in normal control subjects was approximately 1, which is also distinctly different from the values reported in the west where the ratio is close to two [12]. Our data corroborates the observation made by Sehgal *et al *[17] who also documented a lower CD_4_^+ ^cell number, a higher CD_8_^+ ^cell count and CD_4_:CD_8 _ratio of 1 in healthy North Indians.

Besides CD_4_^+ ^and CD_8_^+ ^cells, NK cells are key cells that deal with virally infected cells. Majority of peripheral blood lymphocytes that mediate NK cytotoxic activity in healthy adults express both CD_16 _and CD_56 _. Using these two NK cell markers, a decrease in NK cell number has been reported in HIV infected individuals [18,19]. In the present study too, the basal mean of NK cells were low in immunosupressed patients compared to patients with high CD_4 _counts and normal control subjects. After HBV vaccination, NK cells in controls increased after each dose unlike in patients with high CD_4 _counts where no significant difference was observed. Patients with low CD_4 _counts showed a significant increase only after the last dose of vaccination (170 vs. 293/mm^3 ^p < 0.05). The present study thus illustrates that NK cell response is also compromised in HIV/AIDS.

### Naïve and memory T cells

In HIV infection, CD_4_^+ ^lymphocytes with the memory phenotype harbor more HIV provirus and hence are candidates for depletion by HIV cytopathic effects following activation and subsequent stimulation of viral replication [20]. Although it was initially believed that phenotypically defined memory cells were selectively decreased in HIV infected patients [21], subsequent studies showed that there is no such decrease in the cell number yet a functional impairment could be demonstrated. The exact cause of this functional impairment is still elusive [22,23].

In the present study naïve cells were significantly lower in patients with severe disease while the number of memory cells was increased in HIV/AIDS patients. In spite of this, HBsAb levels were lower than controls thus confirming a functional defect.

### Cytokine response

In our study, HIV infected vaccinated patients failed to elicit adequate IL-4 response. IL4 and IL-10 are the two key cytokines facilitating antibody response. Klein et al [24] also demonstrated that increased IL-4 levels were seen only in early stages of HIV infection. Similarly Kid et al [25] and Romagnani et al [26] questioned the TH1/TH2 imbalance hypothesis and discussed its limitations. We also observed a significant lowering of basal mean IL-10 levels in HIV infected patients compared to controls (p < 0.01). After vaccination, patients with advanced disease showed a delayed and diminished response of IL-10. Thus both IL-4 and IL-10 responses were suboptimal in HIV positive patients, a finding at variance with that reported by Clerici and Sheara [27]. Hong et al [28] and Fakoya et al [29] also documented that TH2 cytokines were increased in HIV infected subjects. Suboptimal responses of both IL-4 and IL-10 observed with HIV patients could have accounted for weak HBsAb titers in HIV infected patients.

In the present study IFN-γ levels were much higher in controls compared to both the groups of HIV infected patients. Further, the response was delayed in patients and the IFN-γ levels could not match the levels observed in controls indicating a lack of T cell help. IL-12 levels are documented to be low in plasma of HIV infected subjects [30] but we could not document any significant difference in IL-12 levels in different groups.

Our study illustrates that HBsAb titers were highest in controls followed by seropositives and minimum in patients with AIDS. Low CD_4_^+ ^lymphocytes and low TH_2 _type of cytokines i.e., IL-4, IL-10 which promote antibody function and functionally abnormal memory cells possibly accounted for low antibody titers in HIV infected patients.

Diaz et al [31] stressed that the response to HBV vaccine may be transitory in AIDS necessitating progressive monitoring of HbsAbs. In a study conducted by Walch and Morse [32], the response rate in patients with CD_4 _counts of > 500/mm^3 ^was 87% while the response rate dropped to 33% in patients with a CD_4 _count between 200–500/mm^3^. In our study only 6/40 patients had CD_4 _counts of > 500/mm^3^, thus the response rate of 100% in our patients with a CD_4 _count between 200–500/mm^3 ^is encouraging. These patients also gained weight and depicted a rise of CD_4_^+ ^lymphocytes. The response rate in controls receiving the conventional dose was 90%. Tedalli et al [33] recommended that patients with a CD_4 _count of < 200/mm^3 ^should be treated with HAART first and HBV vaccine given preferentially after the cut off of 200/mm^3 ^is achieved. This, however, is not possible in resource poor countries. In an excellent comprehensive study on care of patients with chronic HBV [34], the international panel recommended that the antiHBs titers should be checked 12 weeks after immunization and booster given if the response is sub optimal. A short statement of the first European Consensus Conference on the treatment of patients with HBV and HCV reiterates that all HIV patients should be tested for viral hepatitis including those in under served areas and vaccination/treatment offered as and when necessary [35].

## Conclusion

In our patients with low CD_4 _count, in spite of the double dose of vaccine, only 47% were responders and with poor titers against HBV and patients with CD^4 ^counts of < 50/mm^3 ^did not respond at all. Thus early immunization is advocated in all HIV positive patients at a stage when they are still capable of mounting an adequate immune response.

## Competing interests

The author(s) declare that they have no competing interests.

## Authors' contributions

NP carried out the phenotype and cytokine assays and the main bench work. UD guided (main guide) and provided the funds. YC did the liver workup of patients and HBV ELISAs. SS^3 ^and AS recruited the HIV positive patients for the study. SA was the co-guide in the project. RM and BS helped in the documentation and statistical evaluation of the data. SS^1 ^formulated the project and designed the study.

## Pre-publication history

The pre-publication history for this paper can be accessed here:


